# Supplementary figures and images for: Are you confident enough to act? Individual differences in action control are associated with post-decisional metacognitive bias
Source: PLoS One. 2022 Jun 1;17(6):e0268501. doi: 10.1371/journal.pone.0268501 (PMC9159610; doi:10.1371/journal.pone.0268501)

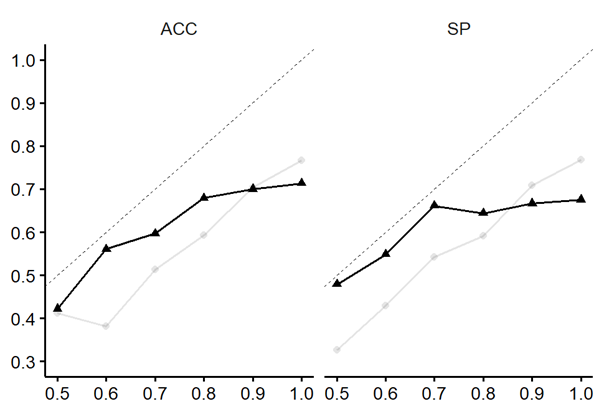

Supplement: S1 Fig — ACC: accuracy emphasis condition. SP: speed emphasis condition. (TIF) [file pone.0268501.s001.tif]

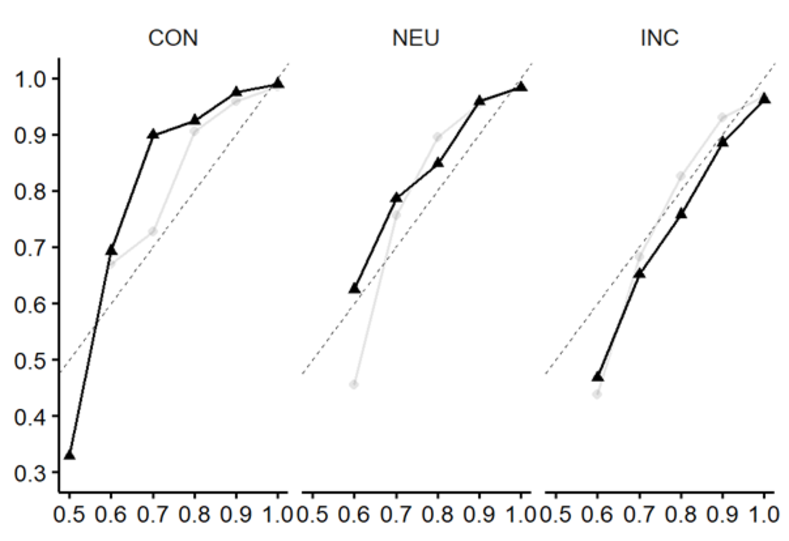

Supplement: S2 Fig — CON: congruent cue condition; NEU: neutral cue condition; INC: incongruent cue condition. (TIF) [file pone.0268501.s002.tif]

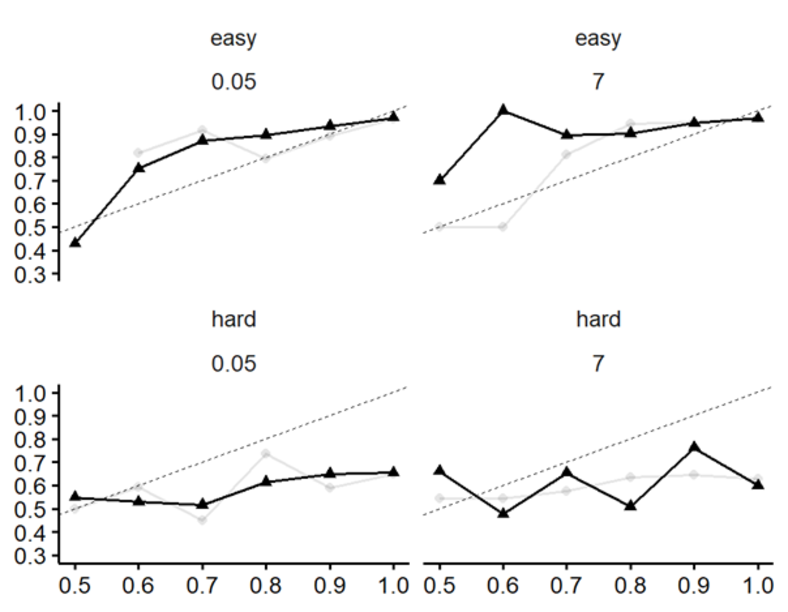

Supplement: S3 Fig — easy: easy condition; hard: difficult condition; 0.05: short choice-confidence interval condition (0.05 sec); 7: long choice-confidence interval condition (7 sec). (TIF) [file pone.0268501.s003.tif]

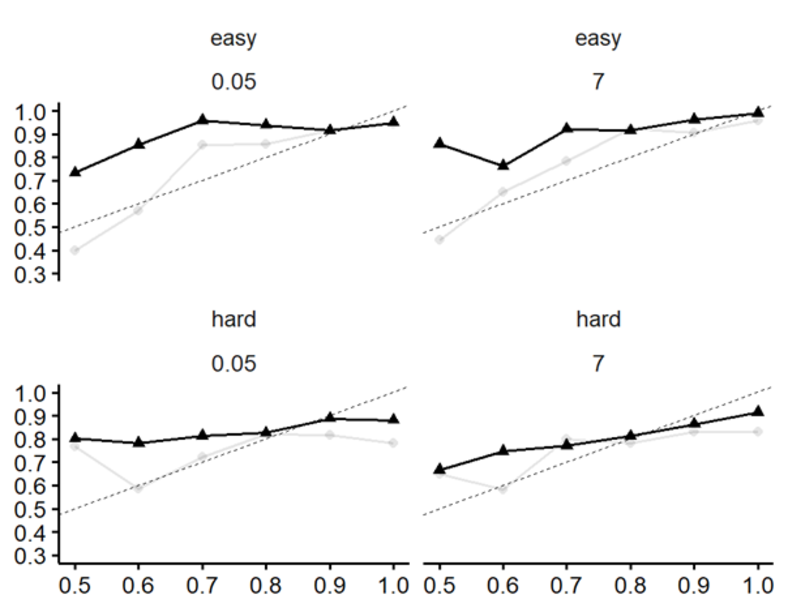

Supplement: S4 Fig — easy: easy condition; hard: difficult condition; 0.05: short choice-confidence interval condition (0.05 sec); 7: long choice-confidence interval condition (7 sec). (TIF) [file pone.0268501.s004.tif]

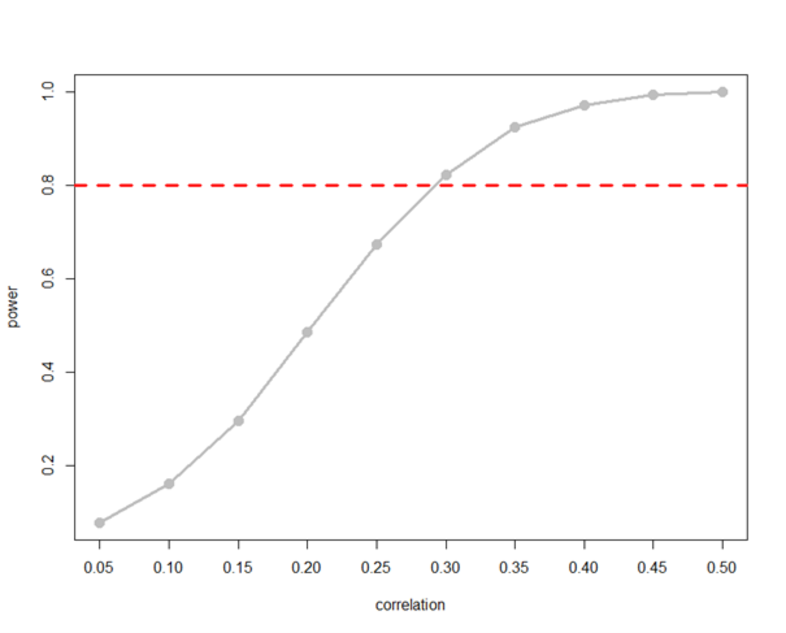

Supplement: S5 Fig — Dashed horizontal red line indicates 80% power level. (TIF) [file pone.0268501.s005.tif]
