# Supplementary material for: Are you confident enough to act? Individual differences in action control are associated with post-decisional metacognitive bias
Source: PLoS One. 2022 Jun 1;17(6):e0268501. doi: 10.1371/journal.pone.0268501 (PMC9159610; doi:10.1371/journal.pone.0268501)
Supplement: S1 Table — (DOCX) [file pone.0268501.s006.docx]

| **RDM Task** | | | | | | | | |
| --- | --- | --- | --- | --- | --- | --- | --- | --- |
|  | Accuracy focus | | | | Speed focus | | | |
|  | Action | | State | | Action | | State | |
| Accuracy (%) | M=67.7 SD=20.0 | | M=66.4 SD=17.3 | | M=65.8 SD=18.5 | | M=64.8 SD=18.0 | |
| RT (ms) | M=727 SD=137 | | M=779 SD=146 | | M=631 SD=140 | | M=665 SD=143 | |
| Confidence (%) | M=89.2 SD=5.4 | | M=82.9 SD=7.7 | | M=86.2 SD=6.6 | | M=80.1 SD=7.3 | |
| **FHD Task** | | | | | | | | |
|  | Congruent | | | Neutral | | Incongruent | | |
|  | Action | State | | Action | State | Action | | State |
| Accuracy (%) | M=92.3 SD=5.7 | M=91.0  SD=6.3 | | M=90.6 SD=7.7 | M=86.8  SD=10.6 | M=87.4  SD=14.1 | | M=78.7  SD=22.0 |
| RT (ms) | M=876  SD=79 | M=885 SD=100 | | M=899 SD=92.5 | M=879 SD=79.2 | M=892 SD=83.7 | | M=903 SD=107 |
| Confidence (%) | M=93.3 SD=5.8 | M=87.9 SD=7.9 | | M=92.5 SD=6.4 | M=87.4 SD=8.6 | M=91.8 SD=7.3 | | M=85.3  SD=9.2 |
