# Supplementary material for: Are you confident enough to act? Individual differences in action control are associated with post-decisional metacognitive bias
Source: PLoS One. 2022 Jun 1;17(6):e0268501. doi: 10.1371/journal.pone.0268501 (PMC9159610; doi:10.1371/journal.pone.0268501)
Supplement: S2 Table — (DOCX) [file pone.0268501.s007.docx]

| **Value-based** | | | | | | | | |
| --- | --- | --- | --- | --- | --- | --- | --- | --- |
|  | Short Interval | | | | Long Interval | | | |
|  | Easy | | Difficult | | Easy | | Difficult | |
|  | Action | State | Action | State | Action | State | Action | State |
| Accuracy (%) | M=93.7 SD=10.6 | M=93.4  SD=13.7 | M=61.1  SD=16.7 | M=59.9 SD=12.9 | M=95.5 SD=9.5 | M=94.9 SD=9.7 | M=62.0 SD=12.6 | M=61.5 SD=12.2 |
| RT (ms) | M=568 SD=109 | M=560 SD=90 | M=656 SD=109 | M=636 SD=110 | M=656 SD=93 | M=563 SD=99 | M=638 SD=121 | M=670 SD=114 |
| Confidence (%) | M=95.8 SD=5.4 | M=92.6 SD=5.6 | M=86.3 SD=9.8 | M=82.1 SD=7.9 | M=95.8 SD=5.0 | M=93.8 SD=4.9 | M=85.9 SD=10.2 | M=80.0 SD=9.1 |
| **Perceptual** | | | | | | | | |
|  | Short Interval | | | | Long Interval | | | |
|  | Easy | | Difficult | | Easy | | Difficult | |
|  | Action | State | Action | State | Action | State | Action | State |
| Accuracy (%) | M=91.2 SD=17.4 | M=92.7 SD=13.2 | M=77.3  SD=14.4 | M=83.9 SD=12.8 | M=92.5 SD=12.6 | M=95.7 SD=9.2 | M=78.9 SD=15.3 | M=79.4 SD=14.8 |
| RT (ms) | M=545 SD=85 | M=564 SD=106 | M=631 SD=113 | M=641 SD=151 | M=546 SD=81 | M=557 SD=101 | M=628 SD=130 | M=641 SD=131 |
| Confidence (%) | M=94.0 SD=5.3 | M=87.9 SD=8.5 | M=86.9 SD=8.4 | M=76.6 SD=11.4 | M=94.1 SD=5.8 | M=90.6 SD=6.8 | M=86.3 SD=9.1 | M=75.4 SD=11.0 |
