# Supplementary material for: Are you confident enough to act? Individual differences in action control are associated with post-decisional metacognitive bias
Source: PLoS One. 2022 Jun 1;17(6):e0268501. doi: 10.1371/journal.pone.0268501 (PMC9159610; doi:10.1371/journal.pone.0268501)
Supplement: S3 Table — (DOCX) [file pone.0268501.s008.docx]

| Variable | *M* | *SD* | 1 | 2 | 3 |
| --- | --- | --- | --- | --- | --- |
|  |  |  |  |  |  |
| 1. RT | 0.75 | 0.14 |  |  |  |
|  |  |  |  |  |  |
| 2. accuracy | 0.67 | 0.09 | .14 |  |  |
|  |  |  | [-.12, .38] |  |  |
|  |  |  |  |  |  |
| 3. confidence | 86.17 | 7.01 | .01 | .17 |  |
|  |  |  | [-.25, .27] | [-.09, .41] |  |
|  |  |  |  |  |  |
| 4. meta-d` | 0.83 | 0.75 | .06 | .72** | .05 |
|  |  |  | [-.21, .31] | [.57, .83] | [-.22, .30] |
|  |  |  |  |  |  |
