# Supplementary material for: Are you confident enough to act? Individual differences in action control are associated with post-decisional metacognitive bias
Source: PLoS One. 2022 Jun 1;17(6):e0268501. doi: 10.1371/journal.pone.0268501 (PMC9159610; doi:10.1371/journal.pone.0268501)
Supplement: S4 Table — (DOCX) [file pone.0268501.s009.docx]

| Variable | *M* | *SD* | 1 | 2 | 3 |
| --- | --- | --- | --- | --- | --- |
|  |  |  |  |  |  |
| 1. RT | 0.65 | 0.14 |  |  |  |
|  |  |  |  |  |  |
| 2. accuracy | 0.65 | 0.10 | .28* |  |  |
|  |  |  | [.03, .50] |  |  |
|  |  |  |  |  |  |
| 3. confidence | 83.27 | 7.42 | .05 | .09 |  |
|  |  |  | [-.21, .31] | [-.17, .34] |  |
|  |  |  |  |  |  |
| 4. meta-d’ | 0.90 | 0.89 | .02 | .70** | .07 |
|  |  |  | [-.24, .28] | [.54, .81] | [-.20, .32] |
|  |  |  |  |  |  |
