# Supplementary material for: Are you confident enough to act? Individual differences in action control are associated with post-decisional metacognitive bias
Source: PLoS One. 2022 Jun 1;17(6):e0268501. doi: 10.1371/journal.pone.0268501 (PMC9159610; doi:10.1371/journal.pone.0268501)
Supplement: S5 Table — (DOCX) [file pone.0268501.s010.docx]

| Variable | *M* | *SD* | 1 | 2 | 3 |
| --- | --- | --- | --- | --- | --- |
|  |  |  |  |  |  |
| 1. RT | 0.88 | 0.08 |  |  |  |
|  |  |  |  |  |  |
| 2. accuracy | 0.92 | 0.05 | -.07 |  |  |
|  |  |  | [-.33, .19] |  |  |
|  |  |  |  |  |  |
| 3. confidence | 90.75 | 6.71 | -.08 | .55** |  |
|  |  |  | [-.33, .19] | [.34, .71] |  |
|  |  |  |  |  |  |
| 4. meta-d’ | 3.86 | 1.19 | .06 | .39** | .41** |
|  |  |  | [-.20, .31] | [.14, .59] | [.17, .61] |
|  |  |  |  |  |  |
