# Supplementary material for: Are you confident enough to act? Individual differences in action control are associated with post-decisional metacognitive bias
Source: PLoS One. 2022 Jun 1;17(6):e0268501. doi: 10.1371/journal.pone.0268501 (PMC9159610; doi:10.1371/journal.pone.0268501)
Supplement: S6 Table — (DOCX) [file pone.0268501.s011.docx]

| Variable | *M* | *SD* | 1 | 2 | 3 |
| --- | --- | --- | --- | --- | --- |
|  |  |  |  |  |  |
| 1. RT | 0.89 | 0.07 |  |  |  |
|  |  |  |  |  |  |
| 2. accuracy | 0.89 | 0.09 | -.00 |  |  |
|  |  |  | [-.26, .26] |  |  |
|  |  |  |  |  |  |
| 3. confidence | 90.07 | 7.40 | -.13 | .65** |  |
|  |  |  | [-.38, .13] | [.47, .78] |  |
|  |  |  |  |  |  |
| 4. meta-d’ | 3.86 | 1.19 | -.09 | .52** | .42** |
|  |  |  | [-.34, .17] | [.30, .69] | [.17, .61] |
|  |  |  |  |  |  |
