# Supplementary material for: Are you confident enough to act? Individual differences in action control are associated with post-decisional metacognitive bias
Source: PLoS One. 2022 Jun 1;17(6):e0268501. doi: 10.1371/journal.pone.0268501 (PMC9159610; doi:10.1371/journal.pone.0268501)
Supplement: S7 Table — (DOCX) [file pone.0268501.s012.docx]

| Variable | *M* | *SD* | 1 | 2 | 3 |
| --- | --- | --- | --- | --- | --- |
|  |  |  |  |  |  |
| 1. RT | 0.90 | 0.08 |  |  |  |
|  |  |  |  |  |  |
| 2. accuracy | 0.83 | 0.18 | .04 |  |  |
|  |  |  | [-.22, .30] |  |  |
|  |  |  |  |  |  |
| 3. confidence | 88.75 | 7.79 | -.17 | .67** |  |
|  |  |  | [-.41, .10] | [.50, .79] |  |
|  |  |  |  |  |  |
| 4. meta-d’ | 3.86 | 1.19 | .07 | .52** | .40** |
|  |  |  | [-.20, .32] | [.31, .69] | [.15, .60] |
|  |  |  |  |  |  |
