# Supplementary material for: Are you confident enough to act? Individual differences in action control are associated with post-decisional metacognitive bias
Source: PLoS One. 2022 Jun 1;17(6):e0268501. doi: 10.1371/journal.pone.0268501 (PMC9159610; doi:10.1371/journal.pone.0268501)
Supplement: S8 Table — (DOCX) [file pone.0268501.s013.docx]

| Variable | *M* | *SD* | 1 | 2 | 3 |
| --- | --- | --- | --- | --- | --- |
|  |  |  |  |  |  |
| 1. RT | 0.61 | 0.09 |  |  |  |
|  |  |  |  |  |  |
| 2. accuracy | 0.78 | 0.09 | -.17 |  |  |
|  |  |  | [-.42, .10] |  |  |
|  |  |  |  |  |  |
| 3. confidence | 89.37 | 6.51 | -.11 | .06 |  |
|  |  |  | [-.36, .16] | [-.21, .31] |  |
|  |  |  |  |  |  |
| 4. meta-d’ | 1.66 | 1.22 | .04 | .26* | -.20 |
|  |  |  | [-.22, .30] | [.00, .49] | [-.44, .07] |
|  |  |  |  |  |  |
