# Supplementary material for: Are you confident enough to act? Individual differences in action control are associated with post-decisional metacognitive bias
Source: PLoS One. 2022 Jun 1;17(6):e0268501. doi: 10.1371/journal.pone.0268501 (PMC9159610; doi:10.1371/journal.pone.0268501)
Supplement: S9 Table — (DOCX) [file pone.0268501.s014.docx]

| Variable | *M* | *SD* | 1 | 2 | 3 |
| --- | --- | --- | --- | --- | --- |
|  |  |  |  |  |  |
| 1. RT | 0.59 | 0.10 |  |  |  |
|  |  |  |  |  |  |
| 2. accuracy | 0.86 | 0.12 | -.05 |  |  |
|  |  |  | [-.31, .22] |  |  |
|  |  |  |  |  |  |
| 3. confidence | 86.83 | 8.23 | -.23 | -.12 |  |
|  |  |  | [-.47, .03] | [-.37, .15] |  |
|  |  |  |  |  |  |
| 4. meta-d’ | 1.66 | 1.22 | .07 | .14 | -.10 |
|  |  |  | [-.19, .33] | [-.13, .39] | [-.35, .17] |
|  |  |  |  |  |  |
