# Supplementary material for: Are you confident enough to act? Individual differences in action control are associated with post-decisional metacognitive bias
Source: PLoS One. 2022 Jun 1;17(6):e0268501. doi: 10.1371/journal.pone.0268501 (PMC9159610; doi:10.1371/journal.pone.0268501)
Supplement: S10 Table — (DOCX) [file pone.0268501.s015.docx]

| Variable | *M* | *SD* | 1 | 2 | 3 |
| --- | --- | --- | --- | --- | --- |
|  |  |  |  |  |  |
| 1. RT | 0.56 | 0.09 |  |  |  |
|  |  |  |  |  |  |
| 2. accuracy | 0.94 | 0.08 | -.25 |  |  |
|  |  |  | [-.48, .01] |  |  |
|  |  |  |  |  |  |
| 3. confidence | 93.20 | 5.19 | -.19 | .09 |  |
|  |  |  | [-.43, .08] | [-.18, .34] |  |
|  |  |  |  |  |  |
| 4. meta-d’ | 1.66 | 1.22 | .05 | .30* | -.06 |
|  |  |  | [-.22, .31] | [.04, .52] | [-.32, .20] |
|  |  |  |  |  |  |
