# Supplementary material for: Are you confident enough to act? Individual differences in action control are associated with post-decisional metacognitive bias
Source: PLoS One. 2022 Jun 1;17(6):e0268501. doi: 10.1371/journal.pone.0268501 (PMC9159610; doi:10.1371/journal.pone.0268501)
Supplement: S11 Table — (DOCX) [file pone.0268501.s016.docx]

| Variable | *M* | *SD* | 1 | 2 | 3 |
| --- | --- | --- | --- | --- | --- |
|  |  |  |  |  |  |
| 1. RT | 0.64 | 0.11 |  |  |  |
|  |  |  |  |  |  |
| 2. accuracy | 0.71 | 0.09 | .05 |  |  |
|  |  |  | [-.21, .31] |  |  |
|  |  |  |  |  |  |
| 3. confidence | 82.73 | 9.43 | -.18 | -.13 |  |
|  |  |  | [-.42, .09] | [-.38, .13] |  |
|  |  |  |  |  |  |
| 4. meta-d’ | 1.66 | 1.22 | .07 | .18 | -.20 |
|  |  |  | [-.20, .32] | [-.09, .42] | [-.44, .07] |
|  |  |  |  |  |  |
