# Supplementary material for: Are you confident enough to act? Individual differences in action control are associated with post-decisional metacognitive bias
Source: PLoS One. 2022 Jun 1;17(6):e0268501. doi: 10.1371/journal.pone.0268501 (PMC9159610; doi:10.1371/journal.pone.0268501)
Supplement: S13 Table — (DOCX) [file pone.0268501.s018.docx]

| Variable | *M* | *SD* | 1 | 2 | 3 |
| --- | --- | --- | --- | --- | --- |
|  |  |  |  |  |  |
| 1. RT | 0.60 | 0.10 |  |  |  |
|  |  |  |  |  |  |
| 2. accuracy | 0.83 | 0.07 | -.07 |  |  |
|  |  |  | [-.33, .19] |  |  |
|  |  |  |  |  |  |
| 3. confidence | 88.10 | 7.06 | -.21 | -.06 |  |
|  |  |  | [-.45, .06] | [-.31, .21] |  |
|  |  |  |  |  |  |
| 4. meta-d’ | 1.66 | 1.22 | .08 | .24 | -.16 |
|  |  |  | [-.19, .33] | [-.02, .47] | [-.40, .11] |
|  |  |  |  |  |  |
