# Supplementary material for: Are you confident enough to act? Individual differences in action control are associated with post-decisional metacognitive bias
Source: PLoS One. 2022 Jun 1;17(6):e0268501. doi: 10.1371/journal.pone.0268501 (PMC9159610; doi:10.1371/journal.pone.0268501)
Supplement: S14 Table — 1) We simulate a variable of interest, correlated with the ACS scale at a given level 2. We randomly selected 60 participants from our pool to the 2 extreme groups, with a minimum combined score of 25 for the action-oriented group, and a maximum combined score of 6 for the state-oriented group (similar to the actual groups used in the experiments). 3. We perform a 2-sample t-test, to see if the difference between groups is detectable at a p<0.05 level. We then run 10000 simulations for each level of correlation (from 0.05 to 0.5, in 0.05 increments). Power can be calculated by subtracting the fraction of false positive findings from all significant results, at each level. (DOCX) [file pone.0268501.s019.docx]

| Correlation | Significant result | False positive | **Power** |
| --- | --- | --- | --- |
| 0.05 | 0.117 | 0.494 | **0.078** |
| 0.10 | 0.198 | 0.336 | **0.161** |
| 0.15 | 0.326 | 0.190 | **0.295** |
| 0.20 | 0.505 | 0.096 | **0.485** |
| 0.25 | 0.682 | 0.040 | **0.673** |
| 0.30 | 0.825 | 0.003 | **0.822** |
| 0.35 | 0.926 | 0.001 | **0.925** |
| 0.40 | 0.970 | 0.000 | **0.970** |
| 0.45 | 0.993 | 0.000 | **0.993** |
| 0.50 | 0.999 | 0.000 | **0.999** |
