# Supplementary material for: Are you confident enough to act? Individual differences in action control are associated with post-decisional metacognitive bias
Source: PLoS One. 2022 Jun 1;17(6):e0268501. doi: 10.1371/journal.pone.0268501 (PMC9159610; doi:10.1371/journal.pone.0268501)
Supplement: Attachment — Submitted filename: Response to Reviewers MR.docx [file pone.0268501.s021.docx]

Dear dr Pamucar,

Thank you for your and the reviewers’ attention and comments on our manuscript. We are pleased that the reviewers have found our work of general interest and importance. The specific comments have been helpful and constructive in the revision of the manuscript. We have addressed each of the issues raised by the referees and revised the manuscript accordingly. Below we summarize our response to the comments, as well as the changes implemented in the revision. The line numbering indicated in the responses below relates to the revised manuscript.

Yours sincerely,

Wojciech Zajkowski, Maksymilian Bielecki, Magdalena Marszał-Wiśniewska

**Reviewer 1.**

*Dear Authors, I believe this manuscript version is improved, but I still have several questions and concerns.*

*1) I was pleased to see calibration curves and correlations added in the appendices. However, no interpretations have been offered. Please provide them, especially about your findings, e.g., how accuracy and confidence, confidence and meta-d' and accuracy and meta-d' correlations may have affected your results and their interpretations (when they are high and then they are low).*

We are thankful for the constructive remarks. In order to address these issues, we added the following paragraphs in the Supplementary Materials:

**Regarding the calibration curve figures:**

*‘The calibration patterns are dependent on task type and difficulty. Both groups display characteristic underconfidence in difficult conditions (Figures S1, S3), as well as slight overconfidence when the task is easy (Figures S2, S3 ,S4) [76]. The main between-group difference is the effect of the state-oriented group being overall more under confident when the task is easy, and less overconfident when the task is difficult. The three plots also speak against simpler heuristic explanations, such as the difference between-groups being driven by the propensity of either action-oriented participants using the highest confidence rating, or state-oriented participants using the lowest.’*

**Regarding correlation figures:**

*‘The correlation tables indicate that the relations between variables of interest vary depending on task and condition. The relation between accuracy and confidence is significant only for the FHD task, i.e. the task with the highest level of accuracy. This is not surprising, since the easier the task, the easier it is to assess one’s own performance, and overall (assuming bias averages out) we should expect more accurate actors to display more confidence.*

*Similar pattern can be observed between confidence and meta-d’ measures, where significant correlations can also be found in the easier experimental conditions, suggesting more confident participants were also more metacognitively accurate. Meta-d’ measure also covaries with accuracy in a number of experimental conditions, suggesting type 1 and type 2 performance being associated on a between-person level (i.e., more accurate participants tended to also be better at metacognitive discrimination). ‘*

*2) Is it possible to adjust the formatting of your correlation tables. I'm aware that R produced them, but I wonder whether you can fix the font and appearance.*

To accommodate this request, we changed the correlation plots to standard tables.

*3) I question your main conclusion that "We propose that a high confidence bias might be crucial for the successful realization of intentions in many real-life situations". A high confidence bias is linked to arrogance and not such great life outcomes (see*

*Bruine de Bruin, W., Missier, F. D., & Levin, P. I. (2012). Individual differences in decision-making competence. Journal of Behavioral Decision Making, 25, 329–330.*

*Bruine de Bruin, W., Parker, A. M., & Fischhoff, B. (2007). Individual differences in adult decision-making competence. Journal of Personality and Social Psychology, 92(5), 938.*

*Kleitman, S., Hui, J.SW. & Jiang, Y. (2019). Confidence to spare: individual differences in cognitive and metacognitive arrogance and competence. Metacognition Learning, 14(3), 479–508* [*http://dx.doi.org/10.1007/s11409-019-09210-x*](http://dx.doi.org/10.1007/s11409-019-09210-x)*).*

*Dunning, D., Johnson, K., Ehrlinger, J., & Kruger, J. (2003). Why people fail to recognize their own incompetence. Current Directions in Psychological Science, 12(3), 83–87.*

*Del Missier, F., Mäntylä, T., & Bruin, W. B. (2012). Decision-making competence, executive functioning, and general cognitive abilities. Journal of Behavioral Decision Making, 25(4), 331–351.*

*Please compare and contrast your research results with these studies.*

*The significant limitations of your studies are small sample sizes, a limited selection of your research task, some stats dependency of your metrics, narrow range. Given all these limitations, I believe you should 'soften' your conclusions and consider possible alternative explanations.*

In order to explore the relation between our findings and the research on overconfidence, we added a paragraph in the general discussion

Lines 903 - 917:

*‘It is also important to contrast our findings concerning the confidence gap (and its known positive correlates) with results underlining detrimental effects of excessive confidence, such as those related to arrogance [102] or political radicalization [103]. Extreme levels of overconficence might also lead to faulty assessments, unrealistic expectations and hazardous decisions [104], which suggests the possibility that the relationship between confidence and beneficial real-life outcomes is not monotonous. At the same time, it also might be worth noting that studies focusing on confidence calibration usually do not distinguish between bias and sensitivity [105-107], making the measure susceptible to being influenced by both factors. Detrimental overconfidence requires both – a high bias, i.e. a tendency to display high levels of confidence in general, as well as poor sensitivity, i.e. the inability to change one’s mind in light of contradicting evidence.*

*In addition, in our study, both groups displayed characteristic patterns of overconfidence when the task was easy and underconfidence when it was difficult  [76] (Supplementary Materials) highlighting that the difference was not qualitative, but  quantitative in nature. ‘*

Since the distinction between bias and overconfidence is not obvious we also clarified the sentence mentioned in the abstract, to make it less ambiguous:

Lines 44 - 46:

*‘We propose that a positive confidence bias, coupled with appropriate metacognitive sensitivity, might be crucial for the successful realization of intentions in many real-life situations.’*

*In addition, we added a paragraph in the limitations section, addressing some alternative explanations:*

*Lines 979 - 986:*

*‘Fourthly, given the limited scope of the study (limited sample, set of experimental tasks and measures used), we cannot fully rule-out some alternative explanations. E.g. performance in a controlled laboratory environment might not be ecologically valid and does not always correspond to real-life situations [121]. Another factor is that confidence ratings are based on self-report scale, making it susceptible to additional confounds. In this light, state-oriented individuals might only express lower confidence while not truly experiencing it. Such report bias [122] could be driven by factors including modesty or excessive self-monitoring [123].’*

We also modified the Conclusions section by softening the sentence in the first paragraph:

Lines 1000 - 1002:

*‘Our results suggest that the differences between groups arise not due to how evidence is obtained or strategic adjustments but due to how it is interpreted by a higher-level process relating evidence to subjective confidence.’*

We also added a sentence explicitly stating the necessity of further studies in order to generalize the findings beyond the realm of value-based and perceptual tasks:

Lines 1014 - 1017:

*‘Further research using different tasks and measures is necessary to confirm these speculations and extrapolate the generalizability of our findings to domains outside of perception and value-based decision tasks.’*

*4) the paper is very dense. I think it would benefit from good editing/proofreading.*

Acknowledging this, we have proof-read the paper, correcting many minor issues and making it clearer.

**Reviewer #2**

*Thank you for addressing the feedback and adding the missing details, as well as supplementary analyses to clarify things. I am completely satisfied with the revision and have no more substantial recommendations, except for one: please edit the newly incorporated text fragments for language and readability, e.g.:*

*"All tasks were performed offline [and] took place in a testing lab on the premises of the university." (p. 15)*

*"To account for this, we perform an additional simulation-based analysis (see: Supplementary Materials) and show that we have over 80% power..." (p. 40) -- it is better to explain the steps of the analyses in the past tense ("we perfomed... and found...") and only the conclusions in the present tense.*

*"We found no support for hypotheses 1 to 3, postulating differences in the decision-making process." (p. 25) -- the comma is probably not needed, as it is a reduced relative clause.*

*There are also similar minor issues in other fragments.*

Thank you for pointing out these language issues. We now corrected these, as well as many other ones throughout the text (corrections visible in the Revised Manuscript with Track Changes)
